# The Effect of Root Coating with Titanium on Prevention of Root Resorption in Avulsed Teeth: An Animal Study

**DOI:** 10.22037/iej.2016.10

**Published:** 2016

**Authors:** Azar Heydari, Soodeh Tahmasbi, Mohammadreza Badiee, SeyedSadra Izadi, Fatemeh Mashhadi Abbas, Sepideh Mokhtari

**Affiliations:** a *Endodontist, Private Practice, Tehran, Iran; *; b * Department of Orthodontics, Dental School, Shahid Beheshti University of Medical Sciences, Tehran, Iran; *; c * Dentofacial Deformities Research Center, Research Institute of Dental Sciences, Shahid Beheshti University of Medical Sciences, Tehran, Iran; *; d * Department of Surgery and Radiology, Faculty of Veterinary Medicine, University of Tehran, Tehran, Iran; *; e *Department of Oral and Maxillofacial Pathology, Dental School, Shahid Beheshti University of Medical Sciences, Tehran, Iran; *; f *Dental School, Tehran University of Medical Sciences, Tehran, Iran*

**Keywords:** Avulsion, Root Resorption, Titanium Coating

## Abstract

**Introduction:**

Tooth avulsion is a real dental emergency. If immediate replantation is not performed, the avulsed tooth may be lost due to inflammatory or replacement resorption. This animal study aimed to evaluate the bone response to the titanium coating of the root surface as an artificial barrier, and prevention of resorption of avulsed teeth.

**Methods and Materials:**

This experimental study was conducted on four male dogs. The dogs were randomly divided into two groups for assessment at two and eight weeks. Four teeth were extracted in each animal. The root surfaces of the test group were coated with a titanium layer using the Electron Beam Deposition system. After 24 h, replantation of the teeth was performed. Two animals were sacrificed after two weeks and the remaining dogs were killed after eight weeks. The presence of inflammation, inflammatory resorption, replacement resorption, periodontal regeneration, periapical granuloma and ankylosis were evaluated through histological analyses.

**Results:**

Inflammatory root resorption was not present in any tooth except one tooth in the coated group after eight weeks. Replacement resorption was noted just in three of the non-coated teeth after two weeks and two teeth after eight weeks. The McNemar's test revealed that the frequency of replacement resorption in the non-coated group was significantly higher than the coated group (*P*=0.031).

**Conclusion:**

Based on the results of this study, it seems that coating the root surfaces of avulsed teeth with titanium may control the replacement root resorption.

## Introduction

Avulsion is a dental emergency which is defined as complete dislodgement of a tooth from its socket. It comprises 0.5 to 3% of all dental traumas [1-3]. Incidence of avulsion is the highest among 7 to 9-year old children because the alveolar bone has the lowest resistance against extrusive forces during this age range [4, 5]. The maxillary central incisors are the most commonly involved teeth and their loss significantly compromises esthetics and function [6, 7]. Tooth avulsion results in injury of the pulp and the periodontal tissues. The ideal treatment for an avulsed permanent tooth is its fast replantation in the socket. Under ideal circumstances, complete regeneration of periodontal tissue is expected to occur [8]. However, the prognosis of an avulsed tooth largely depends on the duration of extra-oral time and the storage medium where the avulsed tooth is maintained before replantation. Evidence shows that the best prognosis is achieved when the avulsed tooth is replanted in its socket within 5 min [5, 8, 9]. In many cases, immediate replantation may not be possible due to the severity of trauma, patient’s psychological or systemic conditions or inadequate knowledge of patients or their companions about how to replant the tooth [4, 6, 7]. Inflammatory resorption can damage the entire root structure within a couple of months while it takes 1 to 5 years for the replacement resorption to damage the entire root [10].

Pulp infection and necrosis, periodontal ligament (PDL) severing and cementum destruction, if associated with the periodontal cell loss on the root surface due to long-term dehydration, initiate a very severe destructive inflammatory process leading to tooth loss shortly after replantation. In such cases, the avulsed tooth must be stored in a medium such as Hank’s balanced salt solution, (HBSS, Sigma-Aldrich, St Louis, MO, USA) or milk in order to maintain the viability of PDL cells [8, 11]. For teeth that have been out of their sockets for less than 1 h in no medium or those stored in a suitable medium for less than 4-6 h after avulsion, the treatment protocol includes immediate replantation with special focus on prevention of inflammatory resorption and replacement resorption. When the avulsed teeth are stored in a dry environment for more than 1 h, the treatment goal is prevention of inflammatory resorption and minimizing the speed of replacement resorption. In this situation, the replacement resorption cannot be prevented; it can only be decelerated to some extent [12, 13]. Immersion of avulsed tooth in fluoridated solutions decreases the pace of root replacement resorption to some extent but cannot completely cease it [8, 12, 13]. Some studies have recommended the application of Emdogain (Biora AB Malmo, Sweden) on the root surface to stimulate progenitor cells for periodontal tissue regeneration. However, further studies showed that Emdogain cannot prevent the occurrence of replacement resorption or cease it [8]. 

The same problems exists for auto-transplanted teeth because during the surgical extraction of tooth from the socket and its replantation in another socket periodontal tissue may be injured and inflammatory resorption and replacement resorption of the root may occur [14]. 

The replacement resorption occurs due to the attack of osteoclasts to root dentin and involvement of dental tissues in the process of bone remodeling [12, 13]. Thus, it appears that by applying a coating on the root surface, serving as an artificial barrier, adhesion of osteoclasts to tooth structure and the consequent replacement resorption may be prevented. However, this coating must have specific properties. For instance, it must be resistant to osteoclastic resorption and should be capable of adhering to both the root surface and bone in order to be able to maintain the tooth in the alveolar socket. In addition, it must be biocompatible and capable of tolerating occlusal forces. Titanium is a metal with extensive medical and dental applications due to its optimal biocompatibility and capability of osseointegration [15, 16]. This metal may be applied to the root surface to meet the above mentioned objectives since it can resist osteoclastic resorption and prevent replacement resorption, it has optimal biocompatibility and is capable of osseointegration. 

This animal study aimed to assess this new concept by evaluating the bone response to the titanium coating on the teeth with mature roots; closed apices; more than 60 min extra-oral time and being held in an inappropriate media.

## Materials and Methods

This experimental study was approved by the Ethics Committee of Shahid Beheshti University of Medical Sciences in Iran )Grant No.: 125(. A pilot study was first conducted to assess the possibility of applying such coating on root surfaces and a 150 nm-thick layer of titanium, was successfully placed on the root surfaces. The maintenance and care of experimental animals complied with National Institutes of Health guidelines for the humane use of laboratory animals. Four mixed-breed male dogs with a mean age of 1.5 years and weight of 20-25 Kg with good general and periodontal health (confirmed by a veterinarian), were selected using non-random convenience sampling. The animals were kept in similar conditions for two weeks. After vaccination and parasite control, the dogs were randomly divided into two groups for assessment at two and eight weeks (two dogs per each group) and each were assigned a code. 

For pre-anesthesia sedation, 0.044 mg/Kg atropine sulfate (Dicatro, Amp 0.5mg/mL, Caspian Tamin Pharmacutical, Rasht, Iran) was injected subcutaneously, 0.27 mg/Kg diazepam (Zepadic, Amp 10mg/2mL, Caspian Tamin Pharmacutical, Rasht, Iran) was injected intravenously and 0.2 mg/Kg butorphanol (Butomidor, 10 mLvial, 10mg/mL, Richter Pharma Ag., Austria) was injected intramuscularly. For induction of anesthesia, 5mg/Kg ketamine chloride (Ketamine 10%, vial 50 ml, 100mg/mL, Alfasan, Netherlands) was administered intravenously. 

The oral cavity was rinsed with chlorhexidine mouthwash and then four teeth were extracted in each animal (teeth #2 and 5 from each quadrant of the mandible) and stored in saline (a total of 16 teeth). The remaining PDL tissues were removed from the root surfaces using a periodontal curette. Then Endodontic treatment was done. Using a fissure diamond bur (Diatech, Heerbrugg, Switzerland), access cavities were prepared and working length was determined using a #10 stainless steel K-file (Mani, Tochigi, Japan). For this purpose, the file was introduced into the canal until its tip was visible at the apex. Of this length, 0.5mm was extracted to yield the working length. Cleaning and shaping of the root canals were carried out by stainless steel hand K-files and Gates Glidden drills (Mani, Tachigiken, Japan) using a step back technique. The canals were rinsed with 0.5% sodium hypochlorite, dried with paper points and filled with gutta-percha (Meta Dental Corp., Chungju, South Korea) and AH-26 sealer (Dentsply, De Trey, Konstanz, Germany) using the lateral condensation technique. To assess the quality of work, the teeth were radiographed. The access cavity and the coronal one-third of the canals were sealed with mineral trioxide aggregate (MTA) (Angelus, Londrina, Paraná, Brazil) and it was covered with a moist cotton pellet to allow complete setting of MTA. 

The roots of teeth #2 in the left and #5 in the right quadrants were considered as the test (coated) group and root surfaces were coated with a titanium layer using the Electron Beam Deposition system (DEBS-3T Model, Microelectronics Lab, Malek Ashtar University, Tehran, Iran). This system is designed for the deposition of most metals and non-metals. In the *e*-beam mode of operation a high-intensity electron beam gun (3 to 20 kV) was focused on the target material in recess in a water-cooled copper hearth. Then the electron beam was magnetically directed onto the evaporation which melted locally.

Before coating, the root surfaces were sandblasted with 50-μm aluminum oxide particles and were then cleaned thoroughly using sodium hypochlorite. To create a uniform thickness of coating on the root surfaces, a device was designed for firm fixation of teeth (from their coronal area), which rotated the teeth during the process of coating. The time was adjusted in such a way that at the end, a titanium layer with approximately 150 μm thickness covered the entire root surface. The crowns of the test (coated) and control (non-coated) groups were cut and separated from the roots at the CEJ level by a disc and the separated roots were then UV-sterilized and stored in a sterile container. Twenty-four h after extraction of teeth, the animals were anesthetized again and the oral cavity was rinsed with chlorhexidine mouth rinse. The blood clots formed in the sockets were removed by a sterile curette and the sockets were rinsed with saline. Prior to replantation, non-coated teeth were prepared according to the standard protocol for mature avulsed teeth that have been in a dry environment for more than 2h. These teeth were immersed in 37% phosphoric acid for 5 min, then stored in 8% stannous fluoride (Sultan, Englewood, USA) for 5 min and were then placed in their respective sockets. The adjacent gingiva was sutured over the respective teeth using polyglyconate sutures (Maxon 5.0; Davis and Geck, Danbury, CT, USA) for watertight closure. Coated teeth were also placed in their respective sockets and the mucosa was sutured as described above. To prevent infection, 22 mg/Kg cefazolin (Cefazolin 500, vial 500 mg, Dena pharmaceutical co., Tabriz, Iran) was administered along with the anesthetic agent intravenously and this process was repeated every 8 h for five days. For pain control, 0.2 mg/Kg butorphanol (Butomidor, 10 mL vial, 10mg/mL, Richter Pharma Ag., Austria) was administered intramuscularly every 8 h for two days. Two animals were sacrificed after 2 days and the remaining two were killed after 8weeks by anesthetic overdose. 

The respective segments of the mandible were separated by a bone saw and transferred to a pathology laboratory. The specimens were immersed in 10% formalin for 3 days and decalcified by immersion in 5% nitric acid for 20 days. The teeth were longitudinally sectioned and the sections were prepared for histological analysis. Of each specimen, at least four 5 μm-thick serial sections from center of specimen (or full length of root) were prepared on two slides and stained with Hematoxylin and Eosin and evaluated by a blind pathologist. For histological analysis, all sections were evaluated under a light microscope (E400, Nikon, Japan) at 40×, 100× and 200× magnifications with regard to the presence of inflammatory cells, inflammatory root resorption (-/+), replacement resorption of the root, periodontal regeneration, periapical granuloma and ankylosis. Further images were taken using stereomicroscope (SZX9, Olympus, Tokyo, Japan) under 12.5 and 25× magnifications.

In order to evaluate the effect of titanium coating on the aforementioned variables and considering the correlated nature of data in split mouth design, the McNemar’s test was performed using SPSS software (SPSS version 21.0, SPSS, Chicago, IL, USA).

## Results

The results of microscopic evaluation are illustrated in Table 1. These data show that after two weeks, inflammatory cells were noted in the periapical area of one coated and around the root of one non-coated teeth. At eight weeks, inflammatory cells were present in the periapical area of one non-coated root. 

**Figure 1:**
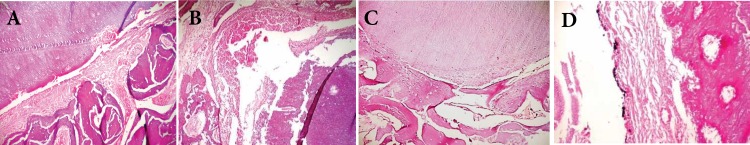
*A) *Periapical granuloma and inflammatory root resorption in a non-coated tooth (25× magnification), *B)* Replacement resorption in the non-coated tooth (12.5× magnification). *C)* Direct contact between secondary cementum and bone following replacement resorption (25× magnification), *D*) A layer of fibrotic tissue surrounding the titanium coated root with no inflammation or active resorption (12.5× magnification

With regard to the inflammatory root resorption, the results were negative for all specimens but one coated tooth after two weeks and one non-coated tooth at eight weeks showed inflammatory root resorption (Figure 1A). Periapical granuloma was noted in one tooth in the coated group after two weeks and in two teeth in the non-coated group after eight weeks. With regard to the replacement resorption, the results were negative in coated teeth at both time points, where as in the non-coated group, replacement resorption was noted in two samples at two weeks and in three after eight weeks (Figure 1B).

No PDL regeneration was seen in the specimens and fibrotic tissue surrounding the root was noted in some samples. At two weeks, connective tissue was noted in three coated and three non-coated teeth. At eight weeks, three teeth in coated group and two teeth in the non-coated group were surrounded by fibrotic tissue (Figure 1C).

Ankylosis without replacement root resorption (the direct contact between tooth and bone) was noted in one tooth in the coated group and one tooth in the non-coated group after eight weeks (Figure 1D). After two weeks, no direct contact between the bone and root was noted.

The McNemar’s test revealed a significant difference between the two groups in terms of the frequency of replacement resorption, while the frequency of replacement resorption in the non-coated group was significantly higher than that in the coated group (*P*=0.031). This analysis revealed no significant difference between the two groups in other parameters at two or eight weeks (*P*>0.05). 

## Discussion

Replacement resorption and inflammatory resorption of root following replantation is the main cause of avulsed tooth loss. Since avulsion mostly occurs in children, preservation of avulsed teeth is very important because if the avulsed tooth is lost, dental implant treatment must be postponed until the child reaches adulthood. Moreover, this time lapse would result in resorption of alveolar ridge, which complicates future dental implant placement. On the other hand, avulsion trauma often occurs in the anterior region involving incisors and therefore, has an adverse effect on the self-confidence and esthetic appearance of the child [9, 12]. If the avulsed tooth remains dry out of the tooth socket for more than 30 min, risk of root resorption significantly increases. Andreasen and Hjorting-Hansen [17] reported that 90% of 110 teeth replanted within 30 min after trauma did not show any inflammatory resorption after 2 years or longer. However, 95% of teeth, which were out of the oral environment for more than 2 h showed inflammatory root resorption. 

Inflammatory root resorption occurs following replantation of avulsed teeth due to necrosis and infection of the pulp and periodontal cells. Elimination of necrotic tissues from the root canal system and its disinfection by calcium hydroxide can prevent the initiation or progression of inflammatory resorption [17, 18]. Thus, in the current study, pulp of all teeth were extracted extra-orally prior to replantation and the root canals were obturated to eliminate the possibility of inflammatory resorption due to pulp infection. Also, the gutta-percha of the coronal root was removed 2 mm below the CEJ level and this area was filled with MTA. This was done due to the higher biocompatibility of MTA and eliminating the possibility of tissue reaction due to using sealer or gutta-percha [19]. Among our specimens, inflammation was noted in only three teeth and inflammatory resorption occurred in one tooth. The probable reason may be the contamination of tooth or socket during the process of replantation. 

In order to decrease the confounding effect of occlusal forces on the bone response to root surfaces, the tooth crowns were cut, the replanted roots were completely covered with mucosa and the two ends of the gingival flap were sutured. It should be mentioned that in real clinical situation decoronation is not possible and the replanted tooth should be splinted to the adjacent teeth [2]. 

Replacement resorption occurs due to trauma to cementum, periodontal cell loss, osteoclasts attacking the root dentin and involvement of dental tissues in the process of bone remodeling [12, 13]. Currently, the available treatment protocols for avulsed teeth emphasize on minimizing the duration of extra-oral time or storing the tooth in an adequate medium during this time period in order to minimize periodontal cell loss [20]. If the above-mentioned conditions cannot be met, the emphasis must be placed on decelerating the process of replacement resorption, because the prevention or cessation of replacement resorption cannot be achieved by the currently available treatments [21, 22]. Several previous studies have evaluated the efficacy of Emdogain for regeneration of periodontal tissues and prevention of root resorption. Guzman and Martinez [23] showed that use of Emdogain alone or in combination with ethylene di-amine tetra-acetic acid (EDTA) did not prevent replacement resorption in dogs. Lam and Sae-Lim [24] reported the same results in monkeys. On the other hand, avulsed teeth, being dry out of the oral environment for more than 1 h, lose all of their periodontal cells and are no longer capable of remaining in bone ,unless the cells and the periodontal tissue regenerate [21, 22]. If this does not happen, the tooth is fused to the surrounding bone. Therefore, ankylosis in these teeth, characterized by the fusion of tooth to the surrounding bone without progressive replacement of root structure with bone, can be an optimal and acceptable outcome. 

**Table 1.** The results of microscopic evaluation

**Time (N)**	**Root ** **condition**	**Inflammatory ** **cells**	**Inflammatory ** **resorption**	**Periapical granuloma**	**Replacement ** **resorption**	**Fibrotic ** **tissue**	**Ankylosis**
**2 weeks (8)**	**Coated**	1	1	1	0	3	0
	**Non-coated**	1	0	0	2	3	0
**8 weeks (8)**	**Coated**	0	0	0	0	3	1
	**Non-coated**	1	1	2	3	2	1
**Total (16)**	**Coated**	1	1	1	0	6	1
	**Non-coated**	2	1	2	5	5	1

Prevention of osteoclast adhesion to root is the key for prevention of replacement resorption [4, 25]. Thus, in the current study, we covered the roots with a titanium coating to serve as an artificial barrier to prevent direct contact of root with the surrounding bone and osteoclasts. Titanium was selected for this purpose because of its unique biocompatibility and osseointegration ability [16, 26], enabling the fusion of titanium-coated root with bone without the occurrence of replacement resorption. Therefore, the current study evaluated the osseointegration of titanium-coated roots and occurrence of root resorption beneath the titanium coating. Since no similar study has been conducted in this regard, we compared our results with those studies that have evaluated the bone tissue response to titanium dental implants to analyze the tissue response to the titanium coating and osseointegration. 

Osseointegration, histologically defined as direct contact of bone with implant, is an essential requirement for a successful dental implant treatment [26, 27]. In our study, ankylosis was defined as direct contact of bone and root without any resorption lacuna in dentin and was considered as the most desirable outcome, which occurred in one tooth in the titanium-coated group and one tooth in the non-coated group. Observation of resorption lacuna containing clastic cells with multiple nuclei in dentin adjacent to the newly formed bone is indicative of replacement resorption in root [19]. This process, in contrast to ankylosis, is progressive and eventually results in replacement of the entire root with bone. This is an undesirable outcome in titanium-coated teeth and histological findings did not report such occurrence in any of the titanium-coated specimens. However, replacement resorption with the above-mentioned pattern was seen in five out of eight non-coated teeth. Therefore, it appears that the titanium coating was successful in preventing the adhesion of dentinoclasts to root surfaces. 

However, presence of soft tissue adjacent to dental implants and absence of a direct contact between the bone and implant are undesirable and indicate treatment failure [21]. As discussed earlier, in titanium-coated group, direct contact of bone with root without resorption was considered as an optimal outcome and was observed in one tooth. In the remaining teeth, a layer of fibrotic tissue was seen between the titanium coating and bone. The reason may be defined as the failure of osseointegration or the difference in the capabilities of the nanometer-scale coating used in our study and that of implants, which makes direct comparison of results impossible. Such coating layer must be resistant to osteoclastic resorption and capable of adhering to the root surface. We could not find a method to quantitatively and objectively assess the adhesion of titanium coating to the root surfaces. Moreover, such coating must be resistant to occlusal forces and this capability must be further scrutinized in future studies.

Further studies are also required to assess the efficacy of other coatings made of other materials such as bioceramics. Finding a suitable coating with the abovementioned criteria can greatly enhance the survival of avulsed teeth and can even enable tooth transplantation in cases of prolonged edentulism (where periodontal regeneration is not possible). Moreover, this method may even have applications for tooth allografts.

It’s worth mentioning that this was a preliminary study with few cases and had many limitations. Additional animal and clinical studies, with more samples, evaluating the bone response by complementary measures such as radiography, are needed to confirm the results of the present study, enabling the clinicians to use the proposed method in clinical situations.

## Conclusion

It seems that coating the root surfaces of avulsed teeth with titanium may control the replacement resorption to some extent.
